# Prevalent role of homologous recombination in the repair of specific double-strand breaks in *Rhizobium etli*

**DOI:** 10.3389/fmicb.2024.1333194

**Published:** 2024-02-28

**Authors:** Fares Osam Yáñez-Cuna, Diana Aguilar-Gómez, Araceli Dávalos, David Romero

**Affiliations:** Programa de Ingeniería Genómica, Centro de Ciencias Genómicas, Universidad Nacional Autónoma de México, Cuernavaca, Mexico

**Keywords:** double-strand break, DNA repair, non-homologous end joining, bacterial recombination, gene conversion

## Abstract

Double-strand breaks (DSBs) are the most dangerous injuries for a genome. When unrepaired, death quickly ensues. In most bacterial systems, DSBs are repaired through homologous recombination. Nearly one-quarter of bacterial species harbor a second system, allowing direct ligation of broken ends, known as Non-Homologous End Joining (NHEJ). The relative role of both systems in DSBs repair in bacteria has been explored only in a few cases. To evaluate this in the bacterium *Rhizobium etli*, we used a modified version of the symbiotic plasmid (264 kb), containing a single copy of the *nifH* gene. In this plasmid, we inserted an integrative plasmid harboring a modified *nifH* gene fragment containing an I-SceI site. DSBs were easily inflicted *in vivo* by conjugating a small, replicative plasmid that expresses the I-SceI nuclease into the appropriate strains. Repair of a DSB may be achieved through homologous recombination (either between adjacent or distant repeats) or NHEJ. Characterization of the derivatives that repaired DSB in different configurations, revealed that in most cases (74%), homologous recombination was the prevalent mechanism responsible for repair, with a relatively minor contribution of NHEJ (23%). Inactivation of the I-SceI gene was detected in 3% of the cases. Sequence analysis of repaired derivatives showed the operation of NHEJ. To enhance the number of derivatives repaired through NHEJ, we repeated these experiments in a *recA* mutant background. Derivatives showing NHEJ were readily obtained when the DSB occurred on a small, artificial plasmid in a *recA* mutant. However, attempts to deliver a DSB on the symbiotic plasmid in a *recA* background failed, due to the accumulation of mutations that inactivated the I-SceI gene. This result, coupled with the absence of derivatives that lost the nonessential symbiotic plasmid, may be due to an unusual stability of the symbiotic plasmid, possibly caused by the presence of multiple toxin-antitoxin modules.

## Introduction

**Figure 1:**
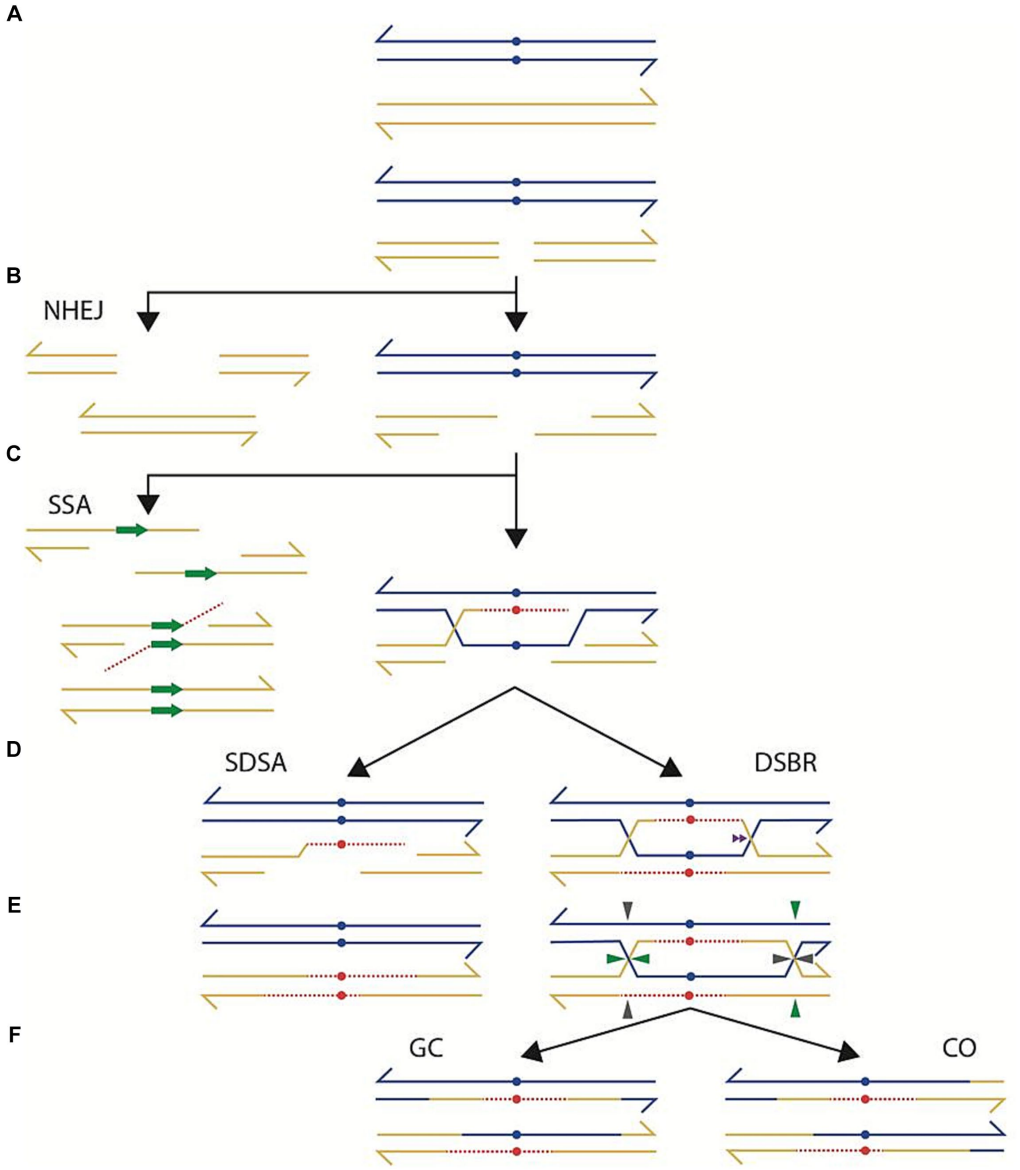
Repair of a double-strand break occurs through a variety of intracellular mechanisms. **(A)** depicts two DNA duplexes, carrying sequence differences (blue dots). One of the duplexes receives a double-strand break, which is processed by degradation (**B**, right part) to generate protruding 3’ends. One of the protruding 3’ends invades the uncut homolog (**C**, right part), synthesizing a complementary strand (interrupted red line) and displacing one of the strands from the uncut homolog. Annealing of the displaced strand with the cut homolog, according to the Double Strand Break Repair model (**D**, DSBR), allows complementary strand synthesis (red), generating a double Holiday junction that can migrate. Resolution of the double Holiday junction **(E)** by cutting in different orientations (horizontal-vertical or vertical-horizontal) generates a repaired product harboring a crossover (CO, **F**) with associated gene conversion. Alternatively, the intermediate in **E** can be cut in the same orientation (either horizontal-horizontal or vertical-vertical), generating a gene conversion product (GC, **F**) without an associated crossover. Other modes of repair of a DSB are also possible. According to the Synthesis-Dependent, Strand Annealing model (SDSA) the intermediate in **C** (right part), after DNA synthesis, may regress to its original location, effectively bridging the degraded sector (**D**, left part). DNA synthesis, using as a template the regressed strand, followed by ligation, generates a repaired product, harboring a gene conversion without an associated crossover. Other repair modes entail a more extensive degradation. In the case of Non-Homologous End Joining (NHEJ), the DSB in **A** is subjected to more extensive degradation (**B**, left), followed by ligation of the resulting ends. Alternatively, the intermediate in **B** (right part) is processed further by single-strand degradation until finding repeated sequences in a direct orientation (**C**, left part). Single-Strand Annealing (SSA) between the repeated regions, followed by gap repair and ligation generates a product with a deletion.

Double-strand breaks (DSB) are a common, yet deadly form of DNA damage; for that reason, bacteria have developed multiple mechanisms for repairing them (Baharoglu and Mazel, 2014). In the presence of either homologous DNA or repeated sequences, most bacteria can repair a DSB faithfully by homologous recombination through a variety of mechanisms (Ayora et al., 2011; Michel and Leach, 2012; Glickman, 2014; Kowalczykowski, 2015; Verma and Greenberg, 2016), depicted in Figure 1. After a DSB (Figure 1A), a helicase-nuclease protein complex resects damaged DNA exposing a 3’ DNA overhang to which the RecA protein binds (Figure 1B). According to double-strand break repair model (DSBR), the RecA protein stimulates the pairing of the 3′ single strand DNA to its homologous sequence and, through DNA polymerization, causes the displacement of the complementary strand (Figure 1C). This strand displacement allows the other 3′ end to anneal to its homolog and polymerize too. This combination of processes ends in the formation of a double Holliday Junction (Figure 1D, DSBR). Resolution of both Holliday Junctions (Figure 1E) generates two possible outcomes: crossover (CO, Figure 1E) or gene conversion (GC, Figure 1E). A variation of this process, called synthesis-dependent strand annealing (SDSA), could also repair a DSB without needing a Holliday Junction. According to this model, DNA resection exposes 3′ overhangs, RecA pairs to the homologous sequences and DNA polymerization, using the homologous strand as a template, effectively bridges the region affected by the DSB (Figures 1A–C). At this point, the polymerized strand stops binding to its homolog and relocates to the other end of the damaged DNA (Figure 1D, SDSA). Since the other end of the damaged DNA has an exposed 3′ end, it can anneal with the newly synthesized DNA. DNA repair terminates with the polymerization of the complementary DNA (Figure 1E, SDSA). An alternative mode of repair, called single-strand annealing (SSA), may operate when a DSB occurs in the interval between two repeated sequences (Tomso and Kreuzer, 2000; Bzymek and Lovett, 2001; Glickman, 2014; Ithurbide et al., 2015). Resection of DNA on both sides of the DSB exposing 3′ ends could leave two single-strand DNA fragments within the repeated sequence (Figure 1C, SSA). Complementarity between the single-stranded repeats allows annealing (Figure 1C, SSA). Repairing the DSB concludes with trimming any single-strand DNA that does not complement each other, thus causing a deletion (Figure 1C). SSA is most prevalent with repeats of small size (< 300 bp) and its operation is reduced upon increasing distance between the repeats (Bzymek and Lovett, 2001, and references therein).

An alternative repair mechanism, prevalent in eukaryotes but present only in 20% of sequenced bacterial genomes (Sharda et al., 2020; Manoj and Kuhlman, 2023), is non-homologous end joining (NHEJ). Some bacteria such as *Bacillus subtilis* (De Vega, 2013), *Pseudomonas putida* (Paris et al., 2015), different *Mycobacterium* (Matthews and Simmons, 2014), *Sinorhizobium meliloti* (Kobayashi et al., 2008 Dupuy et al., 2017), and *Streptomyces ambofaciens* (Hoff et al., 2016) have a set of proteins that allows repair of DSB independently of RecA, through NHEJ. In these bacteria, NHEJ repair depends on a few functions: the recognition of the DSB, done by Ku protein, the remodeling of the 3′ terminus of the DSB, either by adding or deleting nucleotides, performed by the POL and PE domains of LigD, and the joining of broken ends, done by the LIG domain of LigD (Della et al., 2004). As depicted in Figure 1B, NHEJ is a simpler repair mechanism, than can generate deletions or nucleotide insertions.

*Rhizobium etli* is an α-proteobacterium able to fix atmospheric nitrogen in a symbiotic association with bean plants. In *R. etli* CFN42 (the type-strain), the genome comprises three kinds of circular molecules: the main chromosome, one secondary chromosome and five large, dispensable plasmids (González et al., 2006). One of these plasmids, p42d (371.2 kb), contains most of the genes required for nodulation and nitrogen fixation, and was designated as the symbiotic plasmid or pSym. *R. etli* has been shown to do homologous recombination efficiently. Homologous recombination between identical gene copies of the nitrogenase reductase gene (*nifH*) provokes a large deletion in the pSym (pSym_Δ_, 267.7 kb), being a frequent cause of loss of symbiotic abilities (Romero et al., 1991). Similar recombination events between other repeated sequences may also affect symbiotic abilities (Romero et al., 1995). The relative participation of homologous recombination proteins such as RecA (Martínez-Salazar et al., 1991), RecFOR and AddAB (Zuñiga-Castillo et al., 2004), MutS (Santoyo et al., 2005) and RuvB, RecG, and RadA (Martínez-Salazar and Romero, 2000; Castellanos and Romero, 2009; Martínez-Salazar et al., 2009) has already been evaluated in this bacterium, both for crossover formation and gene conversion. Other proteins such as RecQ, RecJ and RecN, among others, have been identified and while its function can be inferred from literature, their participation in DNA repair in *R. etli* has not been assessed yet.

*R. etli* also possesses all proteins required, in other bacteria, to repair DNA by NHEJ. Four copies of the *ku* gene have been identified, two adjacent to each other on the secondary chromosome (named p42e) and two on the main chromosome. Close to the *ku* gene copies there are copies of the *ligD* gene; only one of these (on the secondary chromosome) harbors the LIG, PE and POL domains. This suggests that *R. etli* can repair DSB by NHEJ, although no evidence for this type of repair has been described yet. A similar arrangement was described for the related bacterium *Sinorhizobium meliloti*, where four copies of the *ku* gene and five copies of the *ligD* gene were found (Kobayashi et al., 2008). Mutational analysis in *S. meliloti* revealed that the four copies of the *ku* gene are required for repair of DSBs induced by ionizing radiation treatment, The *sku2* gene copy appears to be the more relevant gene for DSB repair in this bacterium, based on its expression pattern and the substantial reduction in survival of a mutant in this gene after treatment with ionizing radiation (Kobayashi et al., 2008). Interestingly, the NHEJ system in *S. meliloti* appears to contribute to resistance to hydrogen peroxide treatment and desiccation tolerance (Dupuy et al., 2017). Unfortunately, no attempts were made in these studies to characterize how DSBs were repaired. While NHEJ is well-regulated and plays a major role in DNA repair in eukaryotes, in bacteria homologous recombination is the most widespread mechanism for DNA repair. How and when NHEJ participates remains poorly understood. More importantly, the relative participation of NHEJ in the repair of DSB, along with other repair pathways, has been assessed only for a few bacteria, including *Mycobacterium smegmatis* (Gupta et al., 2011), *Pseudomonas putida* (Paris et al., 2015) and *Sinorhizobium meliloti* (Dupuy et al., 2019).

In this work, we are interested in understanding how DSB are repaired on non-essential DNA such as *R. etli* megaplasmids. We evaluate the relative contribution of homologous recombination and no-homologous end joining for repair and characterize the type of events produced by non-homologous end joining.

## Results

### Inducing double-strand breaks on a megaplasmid

To gain insight into the relative role of homologous recombination and NHEJ in *R. etli*, we devised a genetic system, based on the symbiotic plasmid (371 kb) of this bacterium. To facilitate the analysis, a deleted version of the pSym was employed (pSym_Δ_, 268 kb) and cut *in vivo* with an I-SceI nuclease, which recognizes a unique 18 bp site, to generate double-strand breaks on *R. etli* pSym_Δ_.

**Figure 2:**
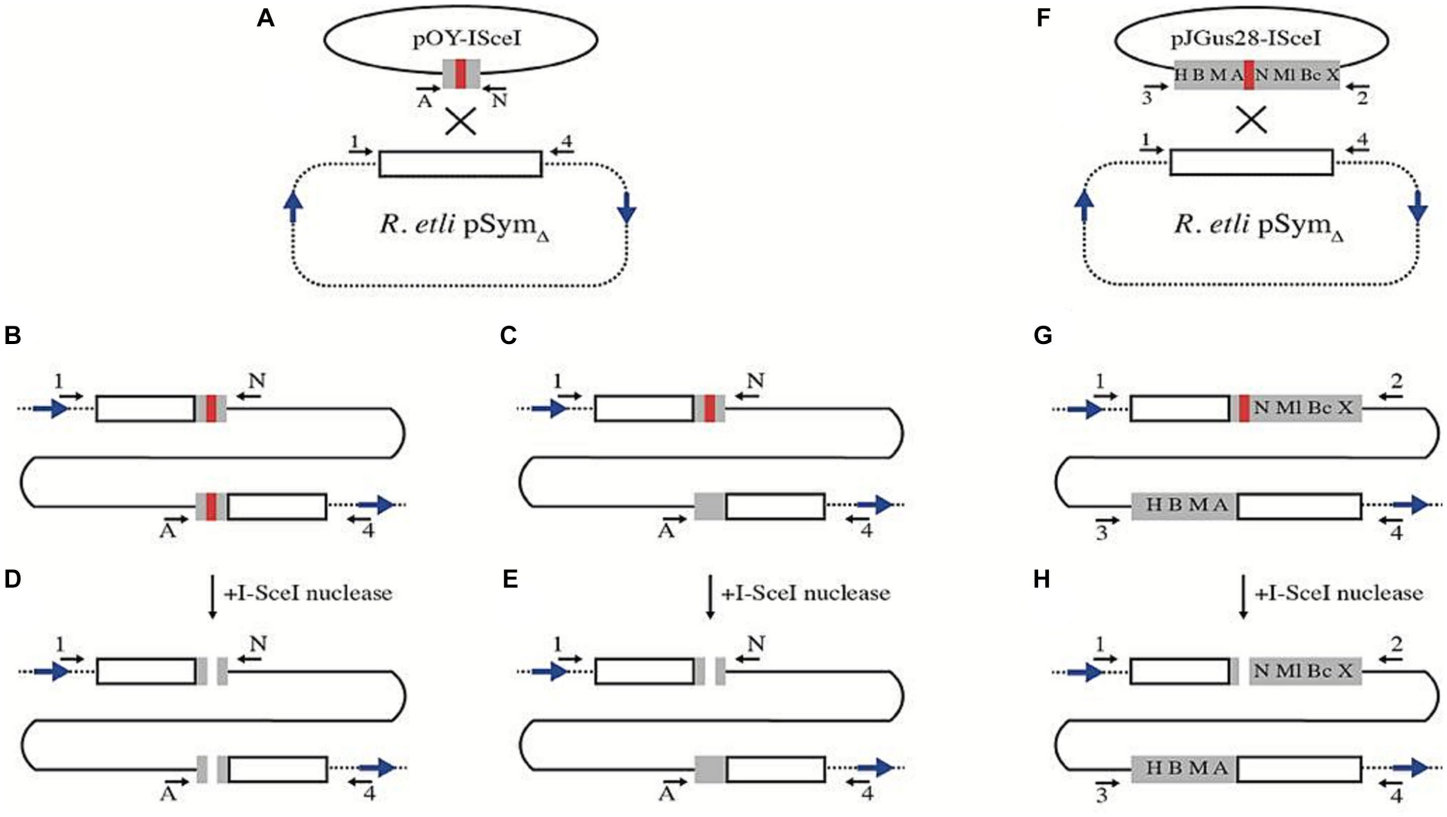
Strategy for the introduction of I-SceI sites on pSym_Δ_. **(A)** Plasmid pOY-ISceI, harboring a 260 bp segment from the *nifH* gene, interrupted by an I-SceI site (red bar) was integrated by homologous recombination into pSym_Δ_. When recombination is accompanied by gene conversion, both duplicated segments of *nifH* contain I-SceI sites (strain CFNX55D2, **B**). Recombination without an associated gene conversion generates a duplication of a segment of *nifH*, with the I-SceI site in one of the duplicates (strain CFNX55D1, **C**). Introduction of the I-SceI nuclease provokes the generation of DSBs either on both **(D)** or in a single *nifH* repeat **(E)**. For introduction of a larger *nifH* repeat, plasmid pJGus28-ISceI was used **(F)**. This plasmid carries a 960 bp sector from *nifH,* containing a series of single-base changes generating restriction sites (H, HindIII; B, BamHI; M, MaeIII; A, ApaLI; N, NarI; Ml, MluI; Bc, BclI; X, XbaI), as well as the I-SceI site (red bar). Recombination without gene conversion on pSym_Δ_ leads to the formation of 960 bp *nifH* repeats, with the I-SceI site in one of the repeats **(G)**. This repeat can be cut upon introduction of the I-SceI nuclease **(H)**. In all panels, blue arrows mark the relative position of F7 repeats, while black arrows (either lettered or numbered) mark the position of PCR primers used for characterization (see Materials and Methods and Supplementary Table S1).

To generate a *R. etli* strain with an I-SceI site on pSym_Δ_, we used plasmid pJGus28-ISceI (Yáñez-Cuna et al., 2016) as a template for amplifying a 260 bp DNA fragment containing the I-SceI site surrounded by 120 bp of the *nifH* gene. This PCR product was then cloned into an integrative plasmid (see Materials and Methods), giving rise to pOY-ISceI. Introduction of this plasmid into *R. etli* harboring pSym_Δ_ generates a cointegrate molecule between pSym_Δ_ and pOY-ISceI (Figure 2). There are two possible outcomes for this cointegration. In the first, non-reciprocal transfer of DNA (gene conversion) results in a cointegrate, with both truncated *nifH* copies now containing the I-SceI site (Figure 2B), giving rise to strain CFNX55D2. In the second, a reciprocal transfer of DNA upon recombination (simple crossover), generates a cointegrate with two truncated versions of the *nifH* gene, one of which harbors the I-SceI site (Figure 2C) giving rise to strain CFNX55D1. To induce a double-stranded break, biparental matings were done between either CFNX55D1 or CFNX55D2 with an *E. coli* carrying plasmid pJN105mega, which expresses the I-SceI gene under the control of the *araBAD* promoter. Isolates recovered after inducing a DSB can be readily classified into homologous recombination, NHEJ and other classes by determinations of plasmid size and amplification with specific primers (Materials and Methods and Supplementary Table S1).

### Repair of DNA breaks with no close homologous regions

Since strain CFNX55D2 contains a small, 260 bp repeated region comprising part of the *nifH* gene and the I-SceI site, expression of the I-SceI nuclease generates two double-strand breaks on pSym_Δ_, with no possibility for the homologous region to be used for repair (Figure 2B). In this case, there are three possible outcomes: (i) an unrepaired pSym_Δ_ could get lost, given that there is no essential DNA on it or, (ii) a DSB could be repaired by NHEJ, leaving a pSym_Δ_ roughly of the same size, or (iii) pSym_Δ_ could be repaired by recombination between distant repeated sequences present on the plasmid. There is ample precedent for recombination events between repeated sequences, other than *nifH*, generating deletions on the pSym (Romero et al., 1995). The most prevalent deletion we obtained, type IV deletion, was generated by recombination between repeated sequences flanking *nifH*, producing a plasmid of 217 kb (Romero et al., 1995). However, the specific sequences involved in the deletion were not characterized at that time. To solve this, a bioinformatic search for nearly identical repeated sequences longer than 100 bp on the pSym was done (Materials and Methods and Supplementary Table S2). The list of repeated sequences was parsed, retaining only those that are in a direct orientation, flanking the *nifH* gene remaining in pSym_Δ_. Recombination between direct repeats of the F7 family (871 bp long, 98% identical) appear to be responsible for generation of type IV deletions.

Introduction by conjugation of plasmid pJN105mega (expressing the I-SceI nuclease) into *R. etli* strain CFNX55 (harboring pSym_Δ_ without I-SceI sites) occurs readily, at a frequency of 7.43 ×10^−4^ (Table 1, line 1). In contrast, when pJN105mega was introduced into *R. etli* CFNX55D2 (harboring pSym_Δ_ with two I-SceI sites), a one-thousand-fold reduction in conjugation frequency was seen (Table 1, line 2), suggesting a large effect of a DSB on transconjugant survival. To characterize the mode of repair of the DSB produced, ten independent biparental matings between *R. etli* CFNX55D2 and *E. coli* S17-I containing pJN105mega plasmid were set up, selecting not more than 5 transconjugants from each mating, to minimize the occurrence of siblings. Detailed characterization of the fifty transconjugants is presented in Supplementary Table S3, while an overview of the results is shown in Table 1.

**Table 1:** Repair after a DNA double-strand break in *R. etli* proceeds through different mechanisms.

Strain	Plasmid introduced	Conjugation frequency (X 10^−4^ ± SD)^a^	Number of repair events by						
			Crossover (distant repeats)	Crossover (adjacent repeats)	GC^b^	NHEJ^c^	Sce-I inactivation	Other	Total
CFNX55	pJN105mega	7.43 ± 2.56	NA^d^	NA	NA	NA	NA	NA	NA
CFNX55D2	pJN105mega	0.005 ± 0.002	46	0	0	0	3	1	50
CFNX55D1	pJN105mega	0.003 ± 0.0007	62	9	6	16	6	1	100
CFNX55O1	pJN105mega	0.005 ± 0.0007	60	11	3	23	3	0	100
CFNX101	pJN105mega	0.24 ± 0.08	NA	NA	NA	NA	NA	NA	NA
CFNX101D2	pJN105	0.45 ± 0.05	NA	NA	NA	NA	NA	NA	NA
CFNX101D2	pJN105mega	0.0003 ± 0.00007	0	0	0	0	46	0	46

a Conjugation frequency is the mean of three independent determinations (CFNX55/pJN105mega, CFNX101/pJN105mega and CFNX101D2/pJN105) or at least ten independent determinations (rest of the data) and are expressed as fraction of transconjugants per recipient cell ± standard deviation (SD).

b GC, gene conversion.

c NHEJ, nonhomologous end joining.

d Not applicable.

None of the isolates characterized presented the loss of pSym_Δ_. In most of the cases (46 out of 50), pSym_Δ_ was reduced in size from 268 kb to 217 kb, coinciding with the plasmid size expected for a type IV deletion. Such a deletion would remove a large segment, including the pOY-ISceI cointegrate and the two I-SceI sites, thus avoiding further events of DSB. To verify that homologous recombination between distant F7 repeats generated this deletion, amplification with appropriate primers was done, evaluating the presence of each individual repeat (F7a and F7b) and the predicted joinpoint (F7a/b, see Materials and Methods). In these 46 cases, no amplification was observed for the individual repeats, but a strong amplification was detected for the predicted joinpoint. Absence of the pOY-ISceI cointegrate and corresponding I-SceI sites was also verified by specific amplification reactions. These results validate that repair of the DSB was achieved by homologous recombination between distant F7 repeats. Of the remaining transconjugants, three of them appear to use a common mechanism for survival. All three have a plasmid matching in size pSym_Δ_; amplifications with appropriate primers revealed that the pOY-ISceI plasmid is still cointegrated and that both I-SceI sites are still present, ruling out NHEJ. To verify the integrity of the I-SceI gene, amplifications were carried out with primers complementary to that gene. In all three transconjugants, this reaction produced a larger PCR product than expected for the nuclease gene, indicating that an insertion had inactivated the function of the nuclease (see below). PCRs from the last transconjugant indicated that a repair process different from the ones described before had happened. PCRs targeting the *nifH* gene and the I-SceI sites did not gave a product, which meant that the digestion had happened yet either the pOY-ISceI plasmid was still cointegrated or that whole region was deleted. The latter being demonstrated by the fact the transconjugant could not grow in kanamycin media. PCRs targeting the F7b repeat generated a product, but the one targeting the F7a repeat, did not. We did not investigate the origin of this derivative any further.

### Repair of DNA breaks with an adjacent homologous region

**Figure 3:**
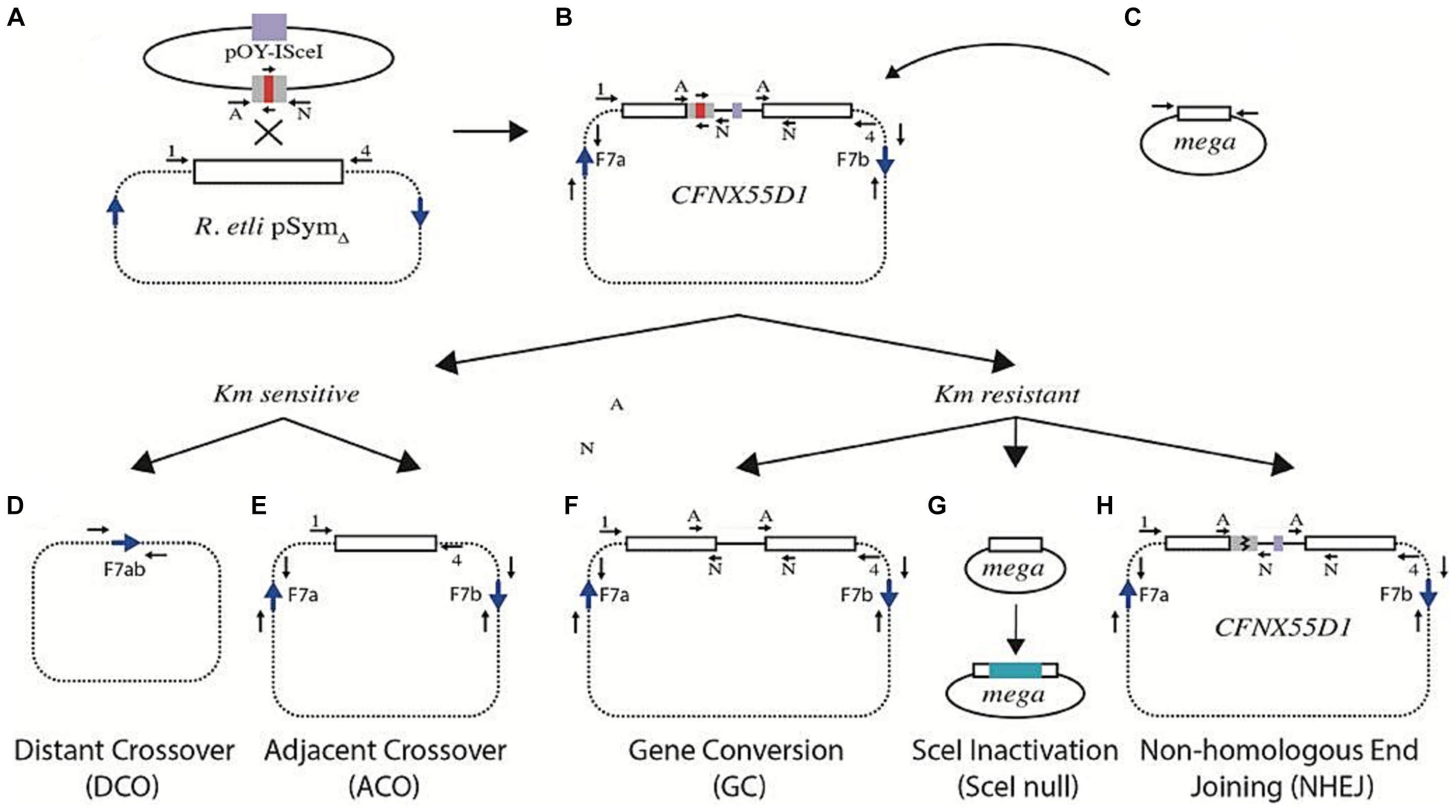
Possible events responsible for repair of a DSB in strain CFNX55D1. **(A)** Plasmid pOY-ISceI [carrying a 260 bp segment from the *nifH* gene interrupted by an I-SceI site (red bar)] was integrated by homologous recombination into pSym_Δ_, leading to introduction of an I-SceI site **(B)**. Upon introduction of a small plasmid carrying the gene for the I-SceI meganuclease **(C)**, a DSB is generated. Events leading to the repair of the DSB include homologous recombination between repeated sequences, either remote (distant crossover, **D**) or contiguous (adjacent crossover, **E**). Both types of events generate derivatives sensitive to kanamycin. Kanamycin resistant derivatives may arise by gene conversion **(F)**, inactivation of the I-SceI gene **(G)**, or non-homologous end joining **(H)**. For all panels, blue arrows mark the relative position of F7 repeats, while small black arrows (either lettered or numbered) mark the position of PCR primers used for characterization (see Materials and Methods and Supplementary Table S1).

As explained before, cointegration of pOY-ISceI into pSym_Δ_ may also generate a strain in which only one truncated *nifH* gene contains an I-SceI recognition site (strain CFNX55D1, Figure 2C). Upon expression of the I-SceI nuclease in this strain, a DSB should occur on the truncated version of the *nifH* harboring the I-SceI site (Figure 2E). Since the other truncated version of the *nifH* gene remains uncut, it is available for recombinational repair of the DSB. The homologous region between the truncated copies of *nifH* is 244 bp long before digestion with I-SceI, and about 119 bp on each side after digestion. Although small, the adjacent homologous region should suffice for recombination, allowing the DNA break repair (Figure 3). Recombination between adjacent *nifH* repeats would occur by simple crossover (leading to excision of the pOY-ISceI cointegrate, Figure 3E) or by gene conversion (maintaining the pOY-ISceI cointegrate but eliminating the I-SceI site by gene conversion using the uncut homolog as a donor, Figure 3F). In this strain, recombination between distant F7 repeats is also possible (Figure 3D), as well as SceI inactivation (Figure 3G) and non-homologous end joining (NHEJ, Figure 3H).

Introduction of a plasmid expressing the I-SceI nuclease (pJN105mega) into *R. etli* CFNX55D1 (harboring pSym_Δ_ with one I-SceI site) occurred at a low frequency (Table 1, line 3). For characterization of the repair mode after inducing a DSB, twenty independent biparental matings between CFNX55D1 and *E. coli* containing pJN105mega plasmid were done, from which 100 randomly selected transconjugants were analyzed (Table 1, detailed information is given in Supplementary Table S4). Seventy-two transconjugants were sensitive to kanamycin, indicating that repair was achieved through homologous recombination, either with distant (Figure 3D) or adjacent (Figure 3E) repeats. Sixty-two of these presented all the hallmarks of repair by homologous recombination between distant F7 repeats (pSym_Δ_ with a size of 217 kb, lack of amplification of F7a and F7b repeats, amplification of a F7a/b joinpoint, absence of the pOY-ISceI cointegrate and I-SceI site, and sensitivity to kanamycin, Figure 3D). Nine of the kanamycin-sensitive transconjugants were classified as being generated by a crossover between adjacent *nifH* repeats, based on a pSym_Δ_ normal size (268 kb) and absence of both the pOY-ISceI cointegrate and I-SceI site (Figure 3E). One of the transconjugants sensitive to kanamycin had a smaller pSym_Δ_ than the others and was not investigated further.

For the remaining 28 kanamycin-resistant transconjugants, all of them showed a normally sized pSym_Δ_. To determine if the I-SceI site was still present, amplifications were carried out with primers that anneal upstream and in the I-SceI site (primers 1 U/ ISceI chK Lw), respectively. Six transconjugants still carried the I-SceI site; amplification of the I-SceI gene in these revealed a larger amplification product, indicating that the I-SceI gene was inactivated by insertion (Figure 3G). To distinguish if the remaining 22 transconjugants were repaired by gene conversion (Figure 3F) or by NHEJ (Figure 3H), a PCR using primers ApaLIU/NarL was set to amplify an area around the expected I-SceI site. Two PCR products were expected because both truncated versions of the nifH gene can be amplified with such primers. If repair was achieved by gene conversion, both products should be of the same size (249 bp); in contrast, repair through NHEJ should reveal two different products, one of 260 bp (corresponding to the one containing an incomplete I-SceI site) and another of 249 bp (from the one lacking the I-SceI site). Results showed that six transconjugants were repaired by gene conversion (Figure 3F), while 16 corresponded to NHEJ events (Figure 3H).

To summarize, in this configuration, homologous recombination accounted for 77% of the repair events observed (62% by recombination between distant repeats, while 15% were by recombination between adjacent repeats, including simple crossover and gene conversion). NHEJ events were detected in 16% of the cases. Other, less frequent events were inactivation of the I-SceI nuclease gene (6%) and one unidentified event.

### Extending the size of an adjacent homologous region in the vicinity of the DSB

**Figure 4:**
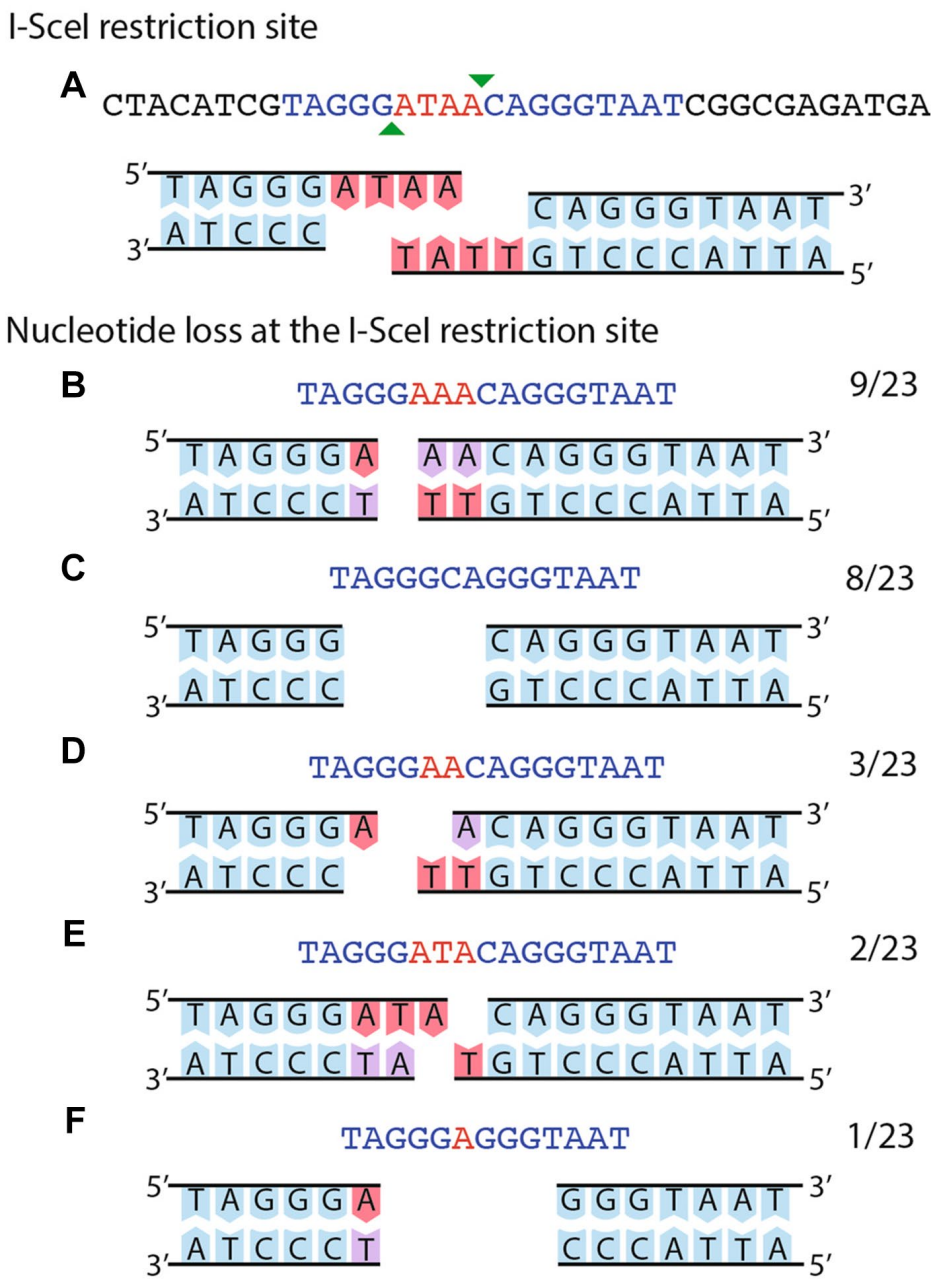
Sequence analysis of NHEJ events obtained in strain CFNX55O1. **(A)** The cut I-SceI site is shown as a double strand sequence. The extent of the I-SceI site is shown in blue, while the protruding ends are shown in red. The bottom part shows the sequence of the top strand. **(B–F)** shows different instances of deletions generated by NHEJ, with the substrate in **(A)** modified to show which nucleotides were deleted. Numbers at the right of each panel show the fraction of events belonging to each class.

Although recombination between distant homologous sequences has been the most prevalent form of DNA repair after induction of DSB, recombination between adjacent homologous sequences in the vicinity of the DSB accounted for 15% of all recombination events. Since the size of the adjacent homologous region available for recombination was small (260 bp), we wanted to test whether a larger homologous region could increase the amount of recombination in the vicinity of the DSB. To increase the size of the homologous region, we employed the integrative plasmid pJGus28-ISceI. This plasmid contains a full copy of the *nifH* gene, with single-base modifications every 100 bp, generating eight different restriction sites. It also has the I-SceI recognition site at the same position as plasmid pOY-ISceI. Cointegration of pJGus28-ISceI into pSym_Δ_ generate a strain with adjacent repeats that are nearly one-kb long, but with the I-SceI recognition site located in only one of the repeats (strain CFNX55O1, Figures 2F–H). As seen before, introduction of a plasmid expressing the I-SceI nuclease (pJN105mega) into *R. etli* CFNX55O1 occurred at a low frequency (Table 1, line 4). For characterization of the repair mode after inducing a DSB, twenty independent biparental matings between CFNX55O1 and *E. coli* containing pJN105mega plasmid were done, from which 100 randomly selected transconjugants were analyzed under the same criteria as before (Table 1, detailed information is given in Supplementary Table S5). Sixty of the transconjugants repaired the DSB by distant crossover between the F7 repeats. Fourteen transconjugants displayed recombination with the extended *nifH* gene copies, of which 11 had an adjacent crossover event, while the remaining three had a gene conversion event. Three transconjugants did not experienced a DSB, due to inactivation of the I-SceI nuclease by an inserted sequence (see below). The remaining 23 transconjugants were repaired by NHEJ. To characterize the extent of NHEJ, sequence of the region surrounding the I-SceI site was obtained for each transconjugant. As shown in Figure 4A, digestion of the I-SceI site generates 4 nt cohesive ends. Sequence analysis of transconjugants showed deletions removing from 1 to 4 nts on the sequence corresponding to the cohesive ends after I-SceI digestion; in one case removal of three nts of the I-SceI site was accompanied by deletion of an extra 2 nt next to the cohesive ends (Figures 4B–F).

### Apparent lethality of a DSB on a *recA* mutant

Since NHEJ is independent of *recA* function, we reasoned that induction of a DSB on a *recA* mutant background would allow us to detect NHEJ events, without interference by homologous recombination. To explore that, the pSym_Δ_ of strain CFNX55D2 was transferred by conjugation into *R. etli* CFNX101 (a *recA* mutant), thus creating strain CFNX101D2. The introduction of a plasmid expressing the I-SceI nuclease into a *recA* mutant background (strain CFNX101) occurred at a ten-fold lower frequency, compared to its introduction in a wild-type background (Table 1, compare rows 1 and 5). A similar effect was observed upon the transfer of pJN105 (a similar plasmid without the nuclease) into strain CFNX101D2. This reduction in conjugation frequency may be explained by the reduced viability of a *recA* mutant. In contrast, when pJN105mega was introduced into *R. etli* CFNX101D2 (*recA* mutant, harboring pSym_Δ_ with two I-SceI sites), a ten-thousand-fold reduction in conjugation frequency was seen (Table 1, row 7), being the lowest conjugation frequency observed in this work.

To characterize the events that allowed survival after a DSB, ten independent biparental matings between strains CFNX101D2 and *E. coli* S17/pJN105mega were carried out, and 46 randomly selected transconjugants were analyzed as before (Table 1). Although we were anticipating the absence of products arising by homologous recombination, to our surprise, no products generated by NHEJ were detected. All the transconjugants showed a normal-sized pSym_Δ_, preserving the pOY-ISceI cointegrate and both I-SceI sites. Analyzing the status of the I-SceI gene by amplification with specific primers, revealed that most of the transconjugants harbor structural alterations in this gene. Thirty-eight of the transconjugants carried insertions (estimated size, 0.7 kb) in the I-SceI gene, while two others had larger insertions, ranging in size from1.4 to 2.1 kb. Two of the transconjugants had deletions in this gene (estimated from 0.1 to 0.15 kb), while four transconjugants carried a normal-sized I-SceI gene. Sequence analysis was done for one of the isolates harboring a deletion, one with an apparently normal I-SceI gene, the two transconjugants carrying the largest insertions and one of the transconjugants bearing a 0.7 kb insertion. A 150-bp deletion was detected, eliminating nt 129 to 279 from the I-SceI gene. For the transconjugant with an apparently normal I-SceI gene, multiple sequence changes were detected (deletion of A73; G > C 429; C > G 654; C > G 660). The largest insertions were caused by incorporation of IS21 (2.1 kb, at nt 249) or IS421 (1.3 kb, at nt 250) into the I-SceI gene; the medium-sized alteration was caused by insertion of IS1A (0.76 kb, at nt 537). Since these insertion sequences originate from the *E. coli* genome (and are absent from *R. etli*) these mutants should have arisen in *E. coli*. Alterations like these were observed previously during this work, but at low frequencies. What is surprising is the predominance of inactivation of the I-SceI gene when a DSB is attempted in a *recA* background. Possible reasons for this are explored in discussion.

### NHEJ was readily observed after induction of a DSB in a small, stable plasmid

Given the inability to induce a DSB on pSym_Δ_ in a *recA* mutant background, we sought alternative ways to explore this issue. For this, we employed plasmid pTR101, a small plasmid containing the origin of replication and the toxin-antitoxin *parDE* operon from plasmid RK2, which has been shown to confer stability in *Sinorhizobium meliloti* (Weinstein et al., 1992). This plasmid lacks extensive repeated sequences. Plasmid pTR101 was modified by introduction of a I-SceI site on it (see Materials and Methods). Both plasmids (pTR101 and pTR101-SceI) were introduced separately into a *recA* mutant background (strain CFNX101). Introduction of pJN105mega by conjugation into strain CFNX101/pTR101 occurred readily (at a frequency of 1 × 10^−5^). In contrast, a strong reduction in transfer frequency of pJN105mega was detected when strain CFNX101/pTR101-SceI was used as a recipient (at a frequency of 1 × 10^−7^).

**Figure 5:**
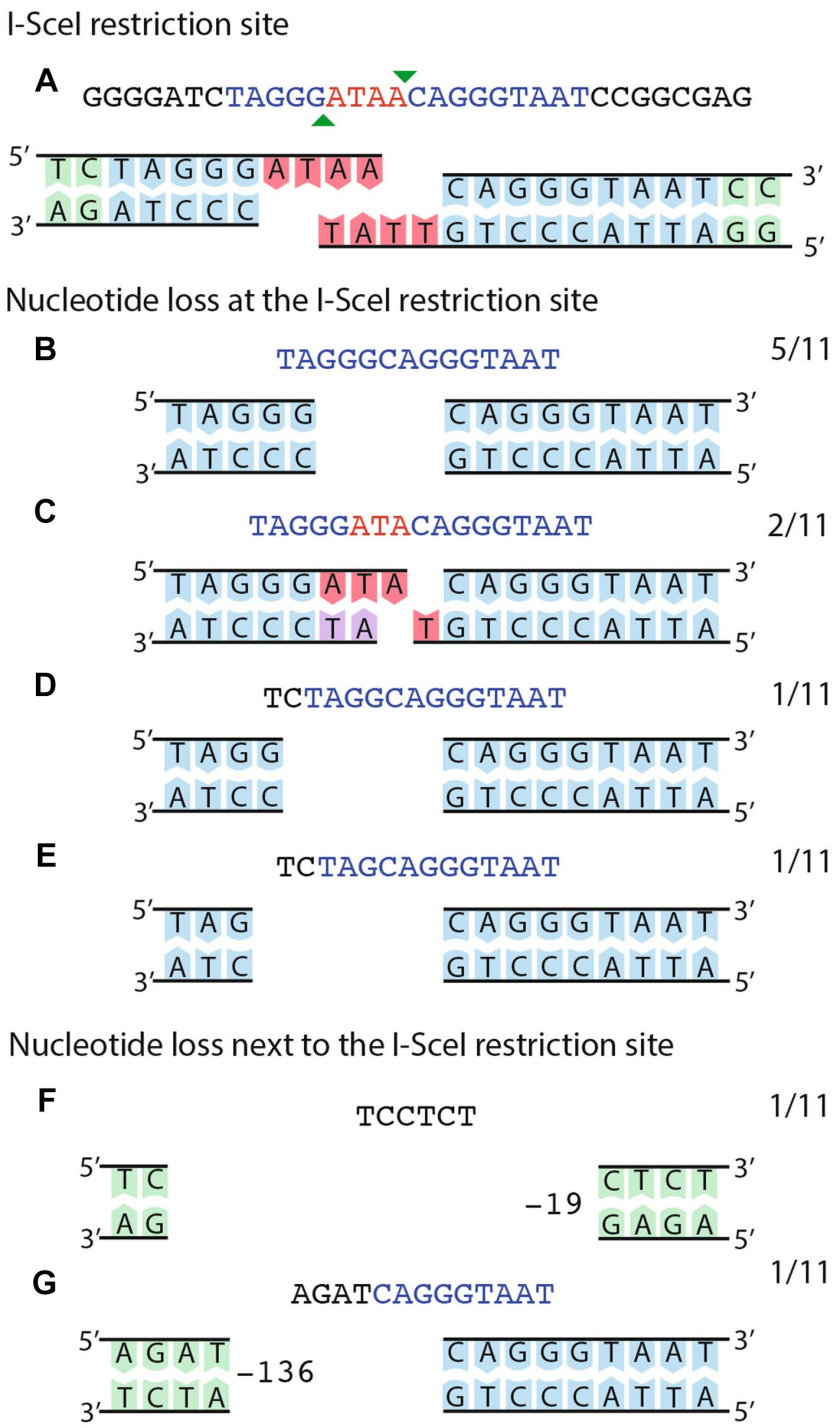
Sequence analysis of NHEJ events obtained on a small, stable plasmid. **(A)** The cut I-SceI site is shown as a double strand sequence. The extent of the I-SceI site is shown in blue, while the protruding ends are shown in red. The bottom part shows the sequence of the top strand. **(B–G)** deletion events generated by NHEJ, either on the I-SceI site **(B–E)** or affecting the I-SceI site and adjacent sequences **(F–G)**. For **F–G**, the number of deleted nucleotides is indicated. Numbers at the right of each panel show the fraction of events belonging to each class.

Sequence analyses of the region surrounding the I-SceI site were obtained for eleven transconjugants of strain CFNX101/pTR101-SceI, harboring pJN105mega (Figure 5). All these showed changes consistent with the operation of NHEJ. Nine of the transconjugants harbored deletions ranging in size from one to six nucleotides, affecting the 5’half of the I-SceI site, while one has a 19 bp deletion that eliminates the I-SceI site. The most extensive deletion removes 136 bp, towards the 5’half of the I-SceI site and adjacent sequences. No microhomologies were detected at the end of these deletions.

## Discussion

The main motivations for this work were to explore the relative contributions of homologous recombination and NHEJ for repairing a double-strand break in *R. etli* and, if NHEJ was detected, to characterize the different events. For that, we employed a system in which either single (strains CFNX55D1 and CFNX55O1) or double (strain CFNX55D2) recognition sites for the I-SceI endonuclease were introduced into pSym derivatives; generation of a DSB was achieved by introduction of a small, self-replicating plasmid harboring the I-SceI gene. Given the configuration and number of I-SceI sites, in strain CFNX55D2 a DSB should be repaired through homologous recombination between distant repeats or by NHEJ, but not by recombination between adjacent repeats. Repair of a DSB in strains CFNX55D1 and CFNX55O1 should be achieved not only by homologous recombination (either between distant or adjacent repeats), but through NHEJ as well (Figure 3). Since the *R. etli* symbiotic plasmid lacks essential genes, we were expecting that the induction of a DSB would not be deleterious for viability.

As expected, repair of DSBs on strain CFNX55D2 was achieved mainly by recombination between distant repeats (92%), with a small contribution of other events (8%), including Sce-I inactivation. We think that NHEJ was not detected in this case due to a small sample size. The relative contribution of homologous recombination and NHEJ is clearly seen in the results obtained with strains CFNX55D1 and CFNX55O1 (Table 1). In these strains, repair by homologous recombination may occur through distant or adjacent repeats, so we combined the abundance of these events. For strain CFNX55D1, 87% of repair events were achieved through homologous recombination, 16% by NHEJ, and 7% by other events, including I-Sce-I inactivation. For strain CFNX55O1 (which contained an adjacent repeat larger than the one in strain CFNX55D1, albeit interrupted by single-base changes), homologous recombination is also the main repair event (74%), compared to NHEJ (23%) or other events (3%). These results support the view that repair of DSBs in *R. etli* is achieved mainly by homologous recombination, being 4 to 5 times more frequent that NHEJ. When repaired by homologous recombination, in both strains recombination between distant repeats was roughly four times more frequent (CFNX55D1, 62%; CFNX55O1, 60%) than recombination between adjacent repeats (CFNX55D1, 15%; CFNX55O1, 14%). We think this difference is due to the size and identity of distant vs. adjacent repeats. Distant repeats are 871 bp long, while adjacent repeats are either 247 bp long (CFNX55D1) or 900 bp long (CFNX55O1). Although in the last case the size of adjacent repeats resembles the size of distant repeats, the adjacent repeats carry single base changes every 100 bp, while distant repeats are identical. We have shown previously that sequence differences limit the use of these repeats for homologous recombination, through the operation of the MutS system (Santoyo et al., 2005).

The prevalence of homologous recombination over NHEJ in the repair of DSB reported here is consistent with data in other systems. In *Mycobacterium smegmatis*, using a chromosomal system where DSBs were generated with I-SceI, DSBs were repaired by homologous recombination in 40% of the cases, by NHEJ (12%) or by a SSA pathway (48%). It was suggested that the SSA pathway may be particularly relevant in repetitive areas of the mycobacterial chromosome (Gupta et al., 2011). For *Sinorhizobium meliloti*, a larger role of homologous recombination over NHEJ in repair of DSBs has also been suggested, based on similar survival rates to ionizing radiation in the wild type and NHEJ mutant strains, while a mutant in *recA* showed a strong reduction in survival (Kobayashi et al., 2008; Dupuy et al., 2017). Interestingly, this difference was observed in growing bacteria, while in stationary phase both homologous recombination and NHEJ contribute to survival from ionizing radiation (Dupuy et al., 2017). A significant contribution of NHEJ was also observed in stationary phase cells of *S. meliloti,* both for repair of DNA ends provided by transformation of linear plasmids or upon delivery of a DSB on the chromosome by *in vivo* digestion with I-SceI (Dupuy et al., 2019).

Repair of I-SceI induced DSBs by NHEJ obtained in this work, either on pSym_Δ_ (strain CFNX55O1) or on a small plasmid (strain CFNX101/pTR101-SceI) were characterized by DNA sequencing. In most of the cases (32 out of 34) deletions, ranging in size from 1–6 bp were observed, centered on the protruding single-strand ends generated by I-SceI digestion. Two cases of larger deletions (19 and 136 bases) were also detected. For repair of a chromosomal DSB in *M. smegmatis* by NHEJ, small deletions (2 to 5 bp) centered on the I-SceI protruding ends were also seen in 7 out of 15 cases; interestingly, the rest of the events carry more extensive deletions, as large as 1,399 bp (Gupta et al., 2011). A similar distribution of deletion sizes was observed for repair events obtained from a *recA* strain. In *S. meliloti*, most of the NHEJ repair events after inflicting a DSB in the chromosome (37 out of 40) were small deletions of 1–4 bp, centered on the protruding ends of the I-SceI site; one of these deletions also exhibited a one-bp insertion. Three cases of large deletions (from 214 to 343 bp) were reported as well (Dupuy et al., 2019). For all three organisms, the main NHEJ repair events are small deletions surrounding the region of the DSB, with varying proportions of larger deletions depending on the organism. The different proportions of large deletions may be due to variability in exonuclease activities between organisms upon delivering a DSB.

An interesting, yet unexpected aspect of our work was the inability to observe derivatives that have lost pSym_Δ_ after delivering a DSB. We were expecting to see this kind of derivatives since sequence analysis reveal the absence of any essential genes on this plasmid (González et al., 2003). Elimination of the pSym of *R. etli* was observed before (Brom et al., 1992, 2000), albeit at a very low frequency (10^−7^). Notably, every time a DSB was inflicted on the pSym_Δ_ a strong reduction in viability (more than a thousand-fold, see Table 1) was observed. A situation like this is commonly observed whenever a DSB is inflicted on a chromosome (Gupta et al., 2011; Dupuy et al., 2019; Uribe et al., 2021), but it is unusual when the DSB occurs on a nonessential plasmid. The inability to eliminate pSym_Δ_, or even to instigate a DSB on it in a *recA* background, can be explained by invoking that, once that homologous recombination (the predominant mode for repair of a DSB) is eliminated, many pSym_Δ_ molecules are left unrepaired. This situation might lead to the elimination of pSym_Δ_, but this could be prevented by the operation of a putative toxin-antitoxin module on pSym_Δ_, killing the cells that have an unrepaired plasmid. Lower frequency alternatives, such as NHEJ, are unlikely to predominate under such circumstances. Under this idea, there should be a strong selection favoring the acquisition of mutations in the I-SceI gene. On the pSym of *R. etli* CFN42 there are at least four potential toxin-antitoxin modules. None of these have been characterized experimentally. Experiments are underway to evaluate the operation of these modules in preventing pSym loss. Interestingly, when a DSB was inflicted on the *E. coli* chromosome using a CRISPR-Cas9 system, a similar frequency of events inactivating Cas9 was observed, whenever the strain is unable to achieve repair by homologous recombination (Uribe et al., 2021).

In conclusion, we have shown that homologous recombination is the main mechanism in *R. etli* for the repair of a DSB. NHEJ is also operational, although it occurs at a four-fold lower frequency. Focusing our work on a nonessential plasmid also revealed an unexpected stability of this plasmid.

## Materials and methods

### Bacterial strains and media

All *Rhizobium etli* strains were grown at 30°C in PY medium (Noel et al., 1984) supplemented with 700 μM CaCl_2_. *Escherichia coli* strains were grown in LB medium at 37°C. Antibiotics were used at the following concentrations (in μg ml^−1^): nalidixic acid, 20; kanamycin, 30; gentamicin 30, spectinomycin 100 and tetracycline 5.

### Plasmid and strain construction

#### Construction of pOY-ISceI

A 247 bp *nifH* gene fragment that includes an I-SceI restriction site was obtained by PCR amplification from pJGus28-ISceI plasmid (Yáñez-Cuna et al., 2016) using primer combination ApaLIU/NarL under the following amplification protocol: 30 cycles of denaturation (94°C, 30 s), annealing (58°C, 30s), and extension (72°C 30s) using *Taq* DNA polymerase (Thermo Scientific). This PCR product was digested with *HindIII* and *XbaI* restriction enzymes and subsequently ligated into a similarly digested pK18mobsacB (Ref) using T4 DNA ligase, giving rise to pOY-ISceI. The ligation mixture was transformed into *E. coli* DH5α [λ^−^ Φ80d*lacZ*ΔM15 Δ(*lacZYA-argF*) *U169 recA1 endA1 hsdR17*(rK^−^ mK^−^) *supE44 thi-1 gyrA relA1*] (Bethesda Research Laboratories, 1986) and transformants were selected in LB media containing kanamycin. Clones were verified by PCR using primers ApaLIU/NarL.

#### Construction of a pTR101 plasmid with I-SceI site

A self-replicating, stable plasmid in Rhizobium, containing the I-SceI site, was constructed using the pTR plasmids (Weinstein et al., 1992). Plasmid pTR101 contains a RK2 stability region, allowing stable maintenance in Rhizobium, even without positive selection. To introduce the I-SceI site into this plasmid, 10 μM of primers SceI site Up and SceI site Lw were annealed on annealing buffer (100 mM Tris pH 7.5, 500 mM NaCl, and 10 mM EDTA) by heating the sample at 95°C for 5 min and then cooled slowly at room temperature. The annealed primers were phosphorylated with T4 Polynucleotide Kinase. Plasmid pTR101 was digested with *BamHI* and treated with Alkaline Phosphatase. Phosphorylated annealed primers were then cloned into pTR101 using T4 DNA ligase, and ligation mixtures were transformed into *E. coli* DH5α, giving rise to pTR101-ISceI. To test that the resulting plasmid had an I-SceI site, a PCR reaction was set up using primer combination SceI site Lw and bla using a protocol of denaturation (94°C, 30s), annealing (55°C, 30s), extension (72°C, 1 m) using *Taq* DNA polymerase. Plasmids pTR101 and pTR101-ISceI were each transformed into an *E. coli* S-17 and then conjugated into *R. etli* CFNX101 on solid PY medium plates (see below). Transconjugants were selected on nalidixic acid and tetracycline.

#### Construction of CFNX55D1 and CFNX55D2 strains

pOY-ISceI was transformed into an *E. coli* S17 (F^−^ *pro-82 thi-1 endA1 hsdR17 supE44 recA13*; chromosomally integrated RP-4-2 [Tc::Mu Km::Tn*7*]) (Simon et al., 1983) and then transferred by conjugation to *R. etli* CFNX55 (Romero et al., 1991). Transconjugants were selected on solid PY medium with nalidixic acid and kanamycin. Since pOY-ISceI does not replicate on *R. etli*, this selects for cointegration with *R. etli* pSym_Δ_ plasmid, interrupting the *nifH* gene. To verify this, a PCR reaction was done using Taq polymerase and primer combination 1 U/4 L, which flanks D plasmid *nifH* gene, under a regimen of 30 cycles of denaturation (94°C, 30s), annealing (48°C, 30s) and extension (72°C, 1m15s). A single crossover event between plasmids generates a cointegrate with only one I-SceI site (CFNX55D1); whereas a gene conversion event, a cointegrate with two I-SceI sites (CFNX55D2). To determine the presence of I-SceI sites at each side of the cointegrate, two PCRs reactions were done with primer combinations 1 U/ISceI chK Lw for one side of the cointegrate and ISceI chK Up/4 L for the other side, using a 30-cycle amplification protocol with Taq polymerase, comprising denaturation (94°C 30s), annealing (44°C, 30s), extension (72°C, 30s). CFNX55D1 and CFNX55D2 strains were also screened for their plasmid profile by the in-gel lysis method of Eckhardt (1978) as modified by Hynes and McGregor (1990).

To generate a strain with one I-SceI site but with a larger homology fragment for recombination, a biparental mating between *R. etli* CFNX55 and *E. coli* S-17 harboring the pJGus28-ISceI plasmid was set up. pJGus28-ISceI plasmid contains a modified version of *R. etli nifH* gene that carries eight single-base pair changes (generating novel restriction sites), and an I-SceI recognition site in the middle of the gene. Transconjugants were selected on solid PY medium with nalidixic acid and kanamycin. Since pJGus28-ISceI does not replicate on *R. etli*, the selection is applied for the cointegration of this plasmid into *R. etli* pSym_Δ_ plasmid. Single crossover recombination between this plasmid and the *nifH* copy on CFNX55 pSym_Δ_ plasmid generates a cointegrate with two full copies of the *nifH* gene. To determine that transconjugants had two copies of the *nifH* gene, a PCR reaction was set up using 1 U/2 L and 3 U/4 L primer combinations with the following PCR amplification protocol: denaturation (94°C, 45 s), annealing (47°C, 45 s), extension (72°C 1 m 15 s) and Taq polymerase. Primers 3 U and 2 L flank *nifH* gene on the pJGus28 plasmid. Transconjugants were also screened for the presence of only one I-SceI site, as described previously, and its plasmid profile, giving rise to CFNX55O1.

#### Construction of CFNX101D2 strain

CFNX101 (Martínez-Salazar et al., 1991) is an *R. etli* CFN42 *recA* strain, to get proof that the *recA* gene is modified, a PCR reaction was set up using primer combination RecAUp/RecALw using the following amplification protocol: 30 cycles of denaturation (94°C, 30s), annealing (55°C, 30s), amplification (72°C, 2m30s) and Taq polymerase. To check for UV sensitivity, CFNX101 was irradiated with UV light at 40 J/m^2^. To transfer our modified pSym_Δ_ plasmid (D2) into a *recA* mutant strain, a biparental mating was done between CFNX55D2 and CFNX101. Selection for transconjugants was done on solid PY medium with spectinomycin and kanamycin. Transconjugants were also screened for the presence of two I-SceI sites using a PCR reaction as described previously. CFNX101D2 strain was also screened for its plasmid profile as described before.

### Molecular characterization of transconjugants

To generate double-stranded breaks at a specific site in the genome, the broad-host-range expression plasmid pJN105meganuclease (Yáñez-Cuna et al., 2016) carrying the gene for the I-SceI nuclease was used. Biparental matings were set up in solid PY medium between *E. coli* S-17 carrying the pJN105meganuclease plasmid and an *R. etli* strain (CFNX55D1, CFNX55D2, CFNX55O1, CFNX101D2). Since pJN105meganuclease plasmid replicates on *R. etli*, the selection causes the transfer of the plasmid into *R. etli* and thus, the expression of the I-SceI nuclease. To reduce the occurrence of siblings in the analysis, 10 independent matings were done for the CFNX55D2 and CFNX101D2 strains and 20, for the CFNX55D1, CFNX55O1, out of which 5 transconjugants were selected randomly for further analyses per mating. Transconjugants were screened for their plasmid profile, as described previously. To determine whether the pOY-ISceI cointegrate had been lost (see Results), transconjugants were grown on solid PY medium with nalidixic acid and kanamycin. DNA from each transconjugant was isolated and subjected to the following analysis: To determine if the repair of DNA involved a crossover event, therefore the pOY-ISceI cointegrate had been lost, we checked for the presence of the *nifH* gene on D plasmid using oligo combinations 1 U/4 L as described before. To determine if D plasmid had a type IV deletion (repair by F7 repeats), we used the following set of three PCR reactions: (1) To screen for the presence of F7 repeat at position 356 Kb we used 356 Fwd/F7 Rev. primer combination; (2) for the presence of F7 repeat at position 202 Kb, we used F7 Fwd/202 Rev. primer combination using the following amplification protocol: denaturation (94°C, 30s), annealing (64°C, 30s), extension (72°C, 1 m) and Taq polymerase; (3) to screen for a recombination event between F7 repeats, a PCR reaction was set up using F7 Fwd/F7 Rev. primer combination using an amplification protocol of denaturation (94°C, 15 s), annealing (68°C, 15 s) and extension (72°C, 1 m) and Taq polymerase. To ensure that a functional I-SceI nuclease had digested DNA at its recognition site, a PCR reaction using primer combination 1 U/ ISceI chK Lw was used, as described before. Since primer ISceI chK Lw anneals into the I-SceI site, point mutations or small deletions generated after DNA repair on the I-SceI site could prevent primer binding and thus, successful PCR. To differentiate between loss of the I-SceI site or changes that prevented the ISceI chK Lw primer to anneal, a PCR was made using ApaLIU/NarL primer combination and *Taq* polymerase as described before, for all transconjugants that had a negative PCR amplification with 1 U/ ISceI chK Lw primers. Moreover, CFNX55O1 pJN105meganuclease transconjugants whose I-SceI site was inferred to be present although with some changes that prevented the PCR from amplifying successfully were sent for sequencing. Primer combination Up 525/Lw 525 was used to screen for the nuclease using the following protocol: denaturation (94°C, 15 s), annealing (60°C, 15 s), extension (72°C, 45 s), and Taq polymerase.

Biparental matings were also set up in solid PY medium between *E. coli* S-17 carrying the pJN105meganuclease plasmid and *R. etli* CFNX101/pTR101 and CFNX101/pTR101-ISceI. Transconjugants were selected on nalidixic acid and gentamycin. To determine if the corresponding pTR plasmid was still present in the transconjugants, these were checked for resistance to tetracycline.

### Estimation of conjugation frequencies

Conjugation frequencies were estimated for all conjugations between an *E. coli* S17-I with plasmids pJN105 or pJN105mega and any *R. etli* strain. Conjugation frequency was calculated as transconjugants between viable recipient cells. After biparental matings were grown for 12 h on solid PY media without antibiotics, cells were resuspended in MgSO_4_ 10 mM and tween 0.01% solution and serially diluted 10-fold to 10–^8^. Dilutions were plated on nalidixic acid for viable recipient cells, and nalidixic acid and gentamicin, for transconjugants. Colonies grown on plates were counted to estimate viable recipient cells and transconjugants. Several conjugation experiments were done for each strain, we report the mean frequency with its standard deviation.

### Bioinformatic tools

REPuter software (Kurtz et al., 2001) was used to search for repeated sequences on *R. etli* p42d. Parameters were set up for finding repeated sequences larger than 100 bp and 99% of identity.

## Data availability statement

The original contributions presented in the study are included in the article/Supplementary material, further inquiries can be directed to the corresponding author.

## Author contributions

FY-C: Formal analysis, Investigation, Writing – original draft. DA-G: Formal analysis, Investigation, Writing – review & editing. AD: Formal analysis, Investigation, Writing – review & editing. DR: Conceptualization, Formal analysis, Funding acquisition, Supervision, Writing – original draft.
